# Quo Vadis Conferences in the Business and Information Systems Engineering (BISE) Community After Covid: What Can Stay, What Should Go, What Do We Need to Change for Our Future Scientific Conferences?

**DOI:** 10.1007/s12599-021-00707-x

**Published:** 2021-07-06

**Authors:** Jan Marco Leimeister, Stefan Stieglitz, Martin Matzner, Dennis Kundisch, Christoph Flath, Maximilian Röglinger

**Affiliations:** 1 University of St, Gallen, St. Gallen, Switzerland; 2 University of Kassel, Kassel, Germany; 3 University of Duisburg Essen, Duisburg, Germany; 4 Friedrich-Alexander University of Erlangen-Nürnberg, Nürnberg, Germany; 5 Paderborn University, Paderborn, Germany; 6 Julius-Maximilians-Universität Würzburg, Würzburg, Germany; 7 University of Bayreuth, Bayreuth, Germany

## Introduction

Jan Marco Leimeister

The global Covid-19 pandemic has not only influenced the way we work and collaborate on our research but also how we present and exchange novel contributions at scientific conferences. Governmental lockdowns and (inter)-national travel restrictions have forced conference organizers to move from offline conference venues to virtual platforms quickly. Our discipline, the Business & Information Systems Engineering (BISE) community, is an interdisciplinary field involving the more conference-driven computer sciences and the more journal-oriented social science. Thus, the impact of Covid-19 on—and the (needed) experience with—new conference formats require a differentiated discourse on the role of the future of our scientific conferences. Hence, we aim to contribute to a discussion on (1) *what can stay offline / as on-site conference formats,* (2) *what we can learn from the experience with (new) online formats,* and (3) *what do we need for our future scientific conferences in the BISE community?*

For more than 350 years, scientific conferences have been the focal occasion to discuss new topics, learn and network for future research. Interested scholars met as early as 1640 to discuss and brainstorm novel research, foster collaborations, and connect with like-minded (Gribbin 2007). Today, scientific conferences across disciplines still aim at the same objectives. According to a survey of over 1000 scientific (offline) conference attendees across fields (Egger and Carpi 2011), 66% of respondents reported that they had learned something that changed the direction of their research, 60% reported that their meeting attendance led to a new collaboration and 51% indicated that something they had learned at the meeting had saved them time and money in their work.

However, in 2020 the organizers of grown conference formats in disciplines such as BISE were forced to rapidly replan and reorganize their scientific exchange formats due to the pandemic. Prominent conferences, such as the *European Conference on Information Systems* (ECIS) in June 2020, the *International Conference on Information System* (ICIS) in December 2020 as well as the *International Conference on Wirtschaftsinformatik* in March 2021, were transformed to virtual events. Surprisingly—from a first perspective—the radical shift to virtual conference formats across disciplines have not only yielded negative organizational effects (e.g., conference organizers needed to move quickly from an offline to an online environment), but have also shown several positive outcomes, such as a general growth in the number of conference participants in various disciplines (i.e., Viglione 2020) or enormous C02 savings of the scientific community worldwide (Viglione 2020). Due to virtual conferences, attendees from around the world were able to access scientific content and discussions regardless of a researcher’s origin, finances, childcare, disabilities, visa or travel restrictions. Moreover, according to a *nature* estimate, virtual conferences led to 45% to 60% less costs, and 80% of scientists answered in June 2020 that conferences should be held virtually, at least to some extent, even after the pandemic subsides (Viglione 2020).

Nevertheless, on second sight, many attendees claim that virtual conferences are not as productive for creative scientific discussions as offline formats. Effects such as "zoom-fatigue", the missing spontaneity of offline events, as well as the continuing daily life at home lead to the subjective impression that solely switching offline to online formats without further innovation and interaction planning is too narrowly considered.

Thus, we aim to initiate a discourse on the future of scientific conferences in the BISE discipline. Or in other words: "Post Covid: What can stay, what should go, what do we need renew for our scientific conferences?".

Besides looking at the short-term effects of Covid-19, we would like to offer the reader a differentiated perspective on conferences in the BISE community. Therefore, we focus on (1) the value proposition of conferences for different stakeholders, (2) the scientific orientation and character(s) of conference contributions in our discipline, and (3) the time and potential for more inclusiveness of conferences and benefits of different (digital) formats:

1. **Value propositions for different conference stakeholder**: Attending BISE conferences, I see not only academics from different career stages but multiple other stakeholders who have a large share in the success and the interdisciplinary character of our meetings. I would at least distinguish between six "personas" with respective goals/"needs" that need to be considered when discussing about conference organization:
  i. Senior researcher (professor) – e.g., aims to network, connect, brainstorm new ideas or recruit
  ii. Researcher (post-doc) – e.g., aims to position their work, network or start collaborations
  iii. Junior (PhD) – e.g., aims to publish, learn, receive feedback or discuss their work
  iv. Newbie (Students) – e.g., aims to experience scientific community, the conference being a pipeline/ motivation for becoming juniors (PhDs)
  v. Practitioner – e.g., aims to network, connect, collaborate or learn
  vi. Panel representative (Journals, SIGs etc.) – e.g., aims to meet, discuss or plan

2. **Scientific orientation of conference contributions in our discipline:** With regards to other disciplines, the significance of conferences often plays diverse roles in the life of a research publication. For example, at *the ACM Conference on Human-Factors in Computing* (CHI), researchers usually publish end-of-life articles (archival), whereas in the *Annual Meeting of the Academy of Management* (AOM), researchers usually send their contribution for feedback in order to revise it for a journal publication. As the BISE community, being an interdisciplinary field including the more conference-driven computer sciences and the more journal-oriented social science, we need to ask ourselves in which direction we want to head and which contributions we want to see presented and discussed at our conferences. Might shorter idea papers be a better way to support the knowledge creation process of our junior researchers instead of long standard literature reviews, where critical scientific discussions are almost impossible?
3. **Time component and benefits of different (digital) formats:** For most stakeholders, conferences do not begin and end with the actual event. Active participants read papers beforehand (*Pre*) and stay in connect for collaboration (*Post*) after a constructive discussion at the conference (*During*). Thinking about the timeline of conferences, offline and online formats bear several advantages, e.g., pre-recorded videos of an online conference enable participants (or even a wider audience) to watch the presentations pre, during and post the event. I also want to highlight innovative formats such as “flipped” conference, in which, similar to “blended learning”, content is consumed alone at home (online), and discussions are conducted in person (offline). In fact, flipped classrooms have been proven to result in higher subjective and objective learning outcomes for participants. A holistic perspective on the conference timeline and on the benefits of different formats (offline, online, hybrid) can only yield advantages for the BISE community.

Triggered by the experience of Covid-19, many associated with BISE are concerned about the future of conferences in our community. While some seem to flirt with virtual formats, others quarrel with them. It is of utmost importance for us to find answers to the questions of *which conference formats can stay offline, what we can learn from the experience with (new) online formats,* and *what needs to be done for our future scientific conferences in the BISE community.* Against this background, we hope that the debaters' positions contribute to an inspiring discourse on the future of scientific conferences in our discipline.

## Video Killed the Conference Star?

Christoph Flath

Conferences play an important role for the scientific community. First and foremost, conferences offer researchers a way to present their most recent work to their peers. At the same time, they connect people on various formal and informal levels, including professional workshops, serendipitous coffee break discussions and memorable conference dinners. Notably, these many personal exchanges render conferences a primary venue for academic recruiting activities. Stepping beyond individual activities, conferences also help establish and maintain a research community’s identity. Major IS conferences (e.g., AIS conferences, the German WI conference) bring together the field thus facilitating the creation of grand visions and positioning statements alike. Conversely, more specialized niche conferences (e.g., DESRIST for design science or BPM for business process management) establish an intimate environment for in-depth collaboration and engagement of the many sub-communities.

The Covid-19 experience has demonstrated that presentations are easily conveyed in an online fashion. At the same time, online conferences unleash numerous benefits – notably no travel emissions, equitable access across different populations, better compatibility with other personal and professional schedules (Viglione 2020). From this one could jump to the conclusion that virtual conferences are better suited to achieve the main goal of academic conferences (presenting research, hosting discussion sessions like the one underlying this position paper, organizing job interviews). When contemplating the virtual conference experience, it seems that the function-first approach reduces conferences to a purely technocratic product losing a warm feeling of community in the process.

Therefore, I do not see a clear dominance of virtual or physical conferences but rather relative advantages in certain areas – virtual is in a certain sense a rational way of running the event (scalable, low individual costs, limited environmental impact) while in-person caters for the emotional side (the power of live encounters, community experience). Consequently, the Covid-19 pandemic may have temporarily removed one way of running conferences but in parallel it has helped us adopt another way (Sein 2020). Going forward and thinking about the future of IS conferences, we need to appreciate having a variety of formats and must acknowledge the underlying dichotomy between rational and emotional events. In turn, we must align the options with the goals associated with individual community events. Following this line of reasoning we should not be surprised to see hybrid event calendars as opposed to hybrid events becoming the norm.

As information systems scholars, we are well-aware of the marked difference between digital transformation and mere digitization of legacy processes (Legner et al. 2017). The latter is markedly shaped by status quo bias and oftentimes will yield sub-optimal results. Given the exogenous shock of switching to the online format without preparation it is understandable that in many cases we replaced the old in-person event in a 1:1 fashion with the digital event – i.e., similar length of the program, comparable number of presentations and clustered with the same ancillary events. This approach corresponds to just doing the same as before. But classic conference formats are a compromise between stakeholder interests and the predominant constraints – cost optimization and space constraints: Fixed costs (travel expenses of participants, setup costs for equipment and venue) stipulate a certain minimum length of an event. Variable costs (accommodation, meals, venue rental fees) limit the length. Similarly, the size and number of rooms limits the number of participants and parallel sessions in turn impact the conference program. From this perspective it seems odd to design online events that mimic offline events as they face a fundamentally different cost structure and constraint set.

The focus should be on establishing formats that complement the standard approach: One-day online events are more plausible than physical events with the same length. This could allow SIG meetings to be uncoupled from conferences and allow them to have multiple meetings per year. The same applies for junior faculty networking or doctoral consortia. The latter could be particularly efficient if a sequence of virtual meetings are crowned by an in-person final event. Similarly, open online seminars have created a level playing field for accessing and presenting the newest research. In general, online formats seem most potent in the context of serving more specialized sub-groups which are only served in a rudimentary fashion by classic conferences (the reason being that they lack the critical mass or budget to hold their own in-person conferences). In this context we should discuss the archival nature of conferences – historically we focus on proceedings, but one could easily see a world where conference videos become the norm, independent of the event taking place in-person or virtually.

While there may not be a single, definitive answer to the future of IS conferences I am convinced that the conference star has not been killed. On the contrary, IS meetings and conferences will emerge in a more varied manner. In this way, the entire community will benefit from a broader range of options.

## Towards Smart Multi-channel BISE Conferences

Maximilian Röglinger

To start with, I am a big fan of conferences in the Business and Information Systems Engineering (BISE) discipline. Most likely, I would not have started my PhD if I had not visited my first BISE conference during my studies where I could listen to all the famous researchers whom I only knew from reading their papers. In the meantime, my personal preferences for attending conferences have changed. Today, I attend about two conferences per year – typically, one general conference such as the BISE conference, ECIS, or ICIS and one special-purpose conference such as the Business Process Management conference. In this discussion, I will sketch my personal view on conference visits and propose three theses on the potential evolution of scientific conferences in the BISE domain.

When I am visiting conferences, I have three things in mind. First, catching up with fellow researchers from the community is most important to me, as conferences create a platform for meeting old and new colleagues both on purpose and by accident. Second, doing community work is my secondary objective, which includes attending editorial board meetings and those of relevant associations. Third, I participate in the scientific program, as conferences enable us to get feedback on preliminary research results and to get in touch with current and future research topics. This is particularly important as topics are evolving so rapidly in the BISE domain that accumulating and sharing knowledge only through journal publications is not enough. So, there is no doubt from my perspective that conferences have played a key role for the successful development of the BISE community and should continue to do so in the future.

However, looking at the exchange formats currently predominating the conference landscape, I feel that we as the BISE community have not tapped the full potential of conferences. Specifically, the scientific main program is rather rigid and deep discussions unfold rarely in the sessions. Despite the undoubted value of conferences, there was room for improvement even prior to the Covid-19 pandemic. One may even question whether the overall output of conferences in their current format justified the huge organizational effort. The Covid-19 pandemic had a twofold effect on conferences. For one thing, the pandemic forced us to experiment with new formats, some of which worked out quite well. These formats enabled much more people to attend conferences and reduced costs for all parties. For another thing, conferences may degenerate into “yet another video conference” and seeing a black wall of participants with their cameras switched off neither fosters academic exchange nor interaction on any other level. Finally, it is hard to get into the flow and to achieve the “mental state of conference” associated with openness for new topics and contacts.

So, the key question is how conferences in the BISE discipline should be set up in the future. To stimulate the discussion on this topic, I would like to present three theses.

- *There will be no purely offline conference anymore*: Analogous to the teaching situation at our universities, there will be no way back to the “old normal”. Like “flipped classrooms”, why not think about “flipped conferences”, i.e., we provide fellow researchers with digital content upfront, skip the traditional presentations, and discuss questions either on site or in hybrid sessions. Thereby, we may reach a bigger audience and increase the depth of scientific discussions. Moreover, participants need not necessarily come to the conference. Rather, the conference comes to them.
- *The “mental state of conference” cannot be reached through entirely digital setups*: In contrast to the virtual components of conferences, we will not achieve the off-site feeling in purely digital setups. For one thing, the daily business too often disrupts the conference schedule. For another thing, spontaneous meetings and new contacts are not sufficiently facilitated through digital platforms. This hampers the exchange within and the further development of the community.
- *Ancillary events will gain in importance*: In the future, the current main and ancillary program may switch roles. We should leverage the interactive and small-group character of the hitherto ancillary formats to increase the output for individual researchers, sub-communities, and the BISE community at large. Exemplary formats that should be used more extensively are doctoral consortia, executive workshops, theory development and paper shepherding workshops, senior scholar speed dating, senior scholar tutorials, paper-a-thons and idea pitch sessions, or meet the editors.

To sum up: If we do not go forwards, we go backwards. The Covid-19 pandemic showed us that changes for the better can be achieved within a short period of time. The overarching question is: *quo vadis scientific conferences*? In light of the theses presented above, specific answers for the BISE community are in high need. This is why we should discuss the pros and cons of different setups – be it offline-only, online-only, or hybrid multi-channel – for individual researchers and the community at large. Most importantly, we should dare to experiment with different setups in order to iteratively converge toward a suitable new normal.

## Why we Should Consider Keeping Virtual Formats for Conferences in Information Systems

Stefan Stieglitz

Hosting the *16th International Conference on Wirtschaftsinformatik* 2021 (WI2021) during the uncertainty of the world-wide Covid-19 pandemic has challenged the conference chairs, Frederik Ahlemann and Reinhard Schütte, and me in many ways. Due to constantly changing news, the conference, which brings together more than 450 researchers, had to be reshaped from the originally planned face-to-face format over a hybrid solution to a fully virtual conference.

When organizing a virtual conference, clear goals need to be defined beforehand. In the context of the WI2021, the aim was to facilitate expert discussions, include workshops and committee meetings, promote networking, and involve practitioners as well as students. Likewise, the conference is intended to maintain and strengthen a community feeling. Moreover, public visibility should be attained to address target groups in the fields of business, media, and politics. Realizing all of these goals in a purely virtual environment raises several issues. These issues represent core subjects of the Information Systems field, such as technology acceptance, design, and adoption as well as data security, digital communication, and collaboration. Nowadays, several platforms and approaches are available that are suitable for achieving the defined goals to a certain degree and tackle the mentioned issues. In this regard, the selection of the platforms and services has to be carefully considered, and the integration of the chosen solutions needs to be well-grounded. With platforms such as Whova and Gather.town, and an accompanying social media strategy on Twitter and Instagram, we were able to pursue the aforementioned goals during the conference, test new approaches, and draw an overall positive conclusion.

When the COVID-19 pandemic is defeated, we could go back to the original face-to-face format in order to conduct the WI conference. However, we could instead rely on our positive experiences of the WI2021 and other virtual conferences, and consider the hybrid approach or the fully virtual format as opportunities for future conferences. Based on the needs and capabilities of the respective conference hosts, the format of the conference could be selected accordingly. Then, the format of the conference could change annually to find the best possible solution for the respective circumstances. In my personal opinion, we should avoid returning permanently to the old status quo without considering new alternatives and their chances. Instead, we need to be open-minded and take a closer look at the newly derived concepts and upcoming opportunities. Since the available platforms have reached a high degree of maturity, virtual formats provide various benefits which I would like to highlight in the following:

- *Environmental protection:* The ecological footprint of virtual conferences is significantly lower than the footprint of events held on-site. Due to the growing awareness of climate change as well as the impetus of the Covid-19 pandemic, avoidable travel could be increasingly criticized in the future. The resulting “Shaming” – which already occurs in the case of airplane travel – could be avoided when hosting a virtual conference.
- *Less expensive:* Virtual conferences are considerably less expensive than face-to-face events. Thus, the conference fee of WI2021 could be significantly reduced compared to previous conferences. In addition, the participants do not have to pay for travel and hotel expenses. Tax money could be saved to a considerable extent.
- *Equal opportunities:* Local conferences require travel, making it more difficult especially for parents to attend the events and present their research articles. Women continue to be more affected by this. The very successful "Women's Lunch" at WI2021 can be seen as an indication that virtual events address the needs of these groups better. Likewise, the higher travel costs of conferences held on-site might be more difficult or impossible for financially poorly endowed chairs to pay, resulting in a possible exclusion of these research groups.
- *Scalability:* Digital formats offer major advantages in terms of scaling. For example, a virtual or hybrid conference could involve more participants without increasing costs to the same extent. As a result, virtual or hybrid conferences could include low-threshold formats for e.g. students or inviting start-ups to certain parts of the conference. It would also be easier to realize requests for current panels or workshops at short notice and therefore address more up-to-date topics, since there would be no need to clarify, for example, room requirements.
- *Participation*: A major goal of conferences is to bring people together. However, in most cases interaction is strongly concentrated on the actual 2–4 days of the (physical) conference. Virtual formats, however, could be used to involve and socially connect PhDs, students, or other stakeholders in all stages of a conference (pre, during, post) even if they cannot attend a physical meeting. Virtual formats could be used by track chairs, associate editors, or reviewers to support online discussions about submitted articles and paper evaluation. Weeks or months before the main conference, students from different universities could jointly work on case studies that are designed and coached by sponsoring enterprises and presented at the conference. After the main conference, virtual spaces could be created to (continuously) develop and improve accepted research papers and RiPs, e.g. by allowing synchronous and asynchronous discussions and by adding new ideas or (updated) references to the documents.
- *Measurability:* Digital formats offer the possibility of (anonymized) data collection and analysis. This can be used to gather information about the topics that move the community (e.g., based on participant numbers, ratings, or text analyses of discussion forums) or to feed recommender systems for conference participants.
- *Persistent content:* If desired, digital content can be made permanently available with only little effort. Then, digital content can be used for further knowledge exchange and acquisition. Moreover, the visibility of the conference and the community can be increased.
- *Motivational concepts:* Virtual platforms nowadays offer functions that are specifically designed to encourage conference visitors to get involved in discussions. Namely, one of these functions is gamification, which was surprisingly popular at WI2021, even without advertising it actively.
- *Business informatics as a pioneer:* Business and Information Systems Engineering / Information Systems defines itself as a leading discipline in the design, management, and analysis of information systems. Thus, for the organization of conferences, the discipline should include information systems which are as professional and strategical as possible in order to exploit advantages and to gain positive visibility towards stakeholder groups.

In contrast to these arguments for a virtual or hybrid conference, several points exist that foster the value of face-to-face conferences, such as richer social proximity and direct discourse, facilitated chance acquaintances, and getting to know different university locations. It would be advisable to see the Covid-19 pandemic as an impetus for weighing all these arguments against each other in order to shape the *International Conference on Wirtschaftsinformatik* in a way that it meets its central goals and find its position for the future.

## The WI Conference can Serve the Purposes of the BISE Community Best if it Remains an In-person Attended Event

Martin Matzner

The BISE community needs a WI conference that is “in good shape”. I would like to share three arguments that are important to me personally (§1–§3). I will then discuss three implications (§4–6) based on my observations.

§1 The WI conference facilitates the development of the set of accepted methods in our community.

Sciences obtain their methods through academic discourse. If we agree on BISE^1^ being different from Information Systems or Business Studies, then this is not only due to the huge sum of individual contributions produced by the BISE community that is displayed for instance at the WI conference. Instead, what makes BISE a research discipline of its own is the collective agreement of its community members on a shared “toolbox” and the joint language that unifies them.

§2 The WI conference gives guidance for “young researchers.”

The conference acts as a compass for the academic job market in the DACH-countries of Germany, Austria, and Switzerland – which comes with many advantages (e.g., excellent “market overview”) and some drawbacks (“cultural and social reproduction”) from the perspective of the discipline. Taking the perspective of an individual young researcher, however, it is important to acknowledge that the key to success in an academic career in the DACH market is not apparent and that the success factors are likely to differ from other disciplines and geographic areas. Especially researchers working abroad or at smaller BISE research institutions (and who therefore have less access to informal information) rely on the informal exchange at the WI conference.

§3 The WI conference is the foundation of the BISE operating model.

Since 1966, BISE has established a superb mix of research, practical orientation, and education that meets a demand. Next to research, many of our colleagues have started successful careers in business, and surprisingly many university boards consist of members from our community.

Today, slogans such as “digitalization” or “digital transformation” permeate our society, and the main themes of 50 years of BISE research receive a lot of attention. This interest also finds expression in the public money spent to expand the related research infrastructure (like Bavaria’s High-Tech-Agenda). However, WI cannot uniquely benefit from that environment, but is challenged by other disciplines such as computer science that increasingly turns towards applications and business research which use “digital” as a default extension to research topics.

The WI conference is needed as a place of “self-reassurance”: Not only for the exposition of BISE’s unique approaches, but also for safeguarding our community’s value propositions from other expanding related fields.

Based on these thoughts, what are important implications for the future of the WI conference?

§4 We should show appreciation and “respect” for our WI conference.

The Thomas “theorem” states that if people define a situation as “real”, then it is “real” in its consequences. It is therefore not sensible to abstain from loudly emphasizing the conference’s strengths. If we acknowledge the important role of the WI conference for the further development of our discipline (e.g., with regard to my previous three propositions), then we all shall be well motivated to commit to the prosperity of the conference: We should acknowledge and appreciate the engagement of our colleagues—in the whole spectrum of our work (as mentor, as reviewer, as member of committees). The conference has many strengths, ranging from “good” quantitative indicators (such as acceptance rates), the diverse and timely set of topics being addressed, and the well-organized events. We should proudly emphasize our conference’s strengths.

§5 We should expand on reflecting and impacting societal debates.

The annual Conference of the German Council of Transport Authorities (“Verkehrsgerichtstag”) in Goslar is never left unmentioned on the daily news reports based on simple instruments. They choose a concrete problem that people can relate to, based on everyday experience. They honor a member of their community to hold an opening speech which the public attention can focus on. Apart from this, a lot of hustle and bustle happens away from the public eye in many working groups. I assume that we could expand the reach of the WI conference using similar instruments.

§6 We should grant the required space for the “grand challenges” of our discipline.

Next to presenting cutting-edge research in detail, an important proposition of the WI conference should be to look at the “broad lines” of our disciplines. In this way, the conference can be a facilitator that helps to focus attention, to cultivate cooperation, to maintain competition, and to provide research funders with an overview (Mertens and Barbian 2015).

### Conclusion

My three arguments for the importance of the WI conference ground in the conventional model of an in-person attended event. They are a consequence of our individual feeling, sense-making, and reshaping of topics, norms, and systems that I cannot imagine to happen in different formats currently. My thoughts on the future development are meant to emphasize that we should not succumb to the danger of limiting our perspective to a “digital” versus “in presence” discussion—as we have plenty of opportunities that we can exploit. I personally hope that a “more digital” WI is a face to face event in essence, when this will be possible in the near future. On behalf of the organizing committee, I would like to invite you to WI’22 in Nuremberg^2^ under the motto “WI for Grand Challenges, Grand Challenges for WI”.

## Thoughts on the Future of the BISE Conference Series

Dennis Kundisch

When I was about to travel to my first BISE conference in Germany, a now-retired colleague told me, “Dennis, you know, a BISE conference is a gathering of our community **interrupted** by research presentations.” Little could he guess the level of disruption to the nature of these gatherings that would be caused by the Covid-19 pandemic, when the organizers of the BISE conference in Duisburg-Essen had to switch to a (mostly) virtual format in 2021.

When reflecting on the value, benefit, and outcomes of such a large annual event, I realized that a BISE conference offers a myriad of reasons and aspects that make up the overall experience of a BISE conference attendance. First and foremost, of course, there are the presentations and discussions of the latest research results, topics, and methods. Moreover, a BISE conference is often used as a platform for co-located workshops, committee meetings, and project meetings. It also offers a supportive environment in which young scientists can practice their skills as presenters and actively engage in the scientific discourse.

But a BISE conference is also so much more: a place not only for the exchange of (scientific) knowledge but also of views and opinions amongst scholars and practitioners. It is an important space for informal consensus-finding within the BISE community. Most of all, it is where the BISE community becomes tangible for all its members: as a networking platform for young and senior researchers, practitioners as well as for students of BISE bachelor and master programs. To local organizers, the conference offers the opportunity to promote their flavor of BISE and use it as an opportunity, through the media coverage, to raise the profile of their home institution and its value to their region. A BISE conference also provides a welcome opportunity to leave the day-to-day routine for a while and immerse yourself in an inspiring experience.

Most of all, the informal spaces it creates for chance encounters between scholars working in the same field (over dinner, or on the bus to dinner, or over a drink at the bar) might end up in unexpected but invaluable outcomes, such as developing research projects with new project partners. In short, there are many reasons that make attending a BISE conference (in its physical form) a worthwhile endeavor; both for the person who experiences it and – at least from my point of view – for the institution that is paying for it.

I envisage at least four (rough) scenarios for the future:

- **“New normal” = “old normal”**: Broadly speaking, we will get back to our usual BISE conference format as soon as it is safe again to do so, simply because so many members of our community are desperately waiting for the moment to finally meet again in person. Further, if some established conference elements were to be replaced with digital formats it could result in a portion of typical participants only joining virtually. As the whole experience is more than just the sum of its individual parts, this could severely erode the positive network effects of the BISE conference, and ultimately lead to its demise in its physical form. Hence, in this scenario conference organizers might be reluctant to offer digital formats and would prefer to return to the “old normal”.
- **Small, but beautiful**: Future BISE conferences with physical presence may become smaller events, because some community members may choose to join in only virtually, and others may have learned in the last months that there is a life outside of attending our annual BISE and other IS conferences (e.g., ICIS, ECIS). In this scenario the basic premise is that network effects are only of secondary importance to a successful, physical BISE conference and that the value of personal attendance by far outweighed the associated direct and indirect costs for most participants in the last years. Hence, replacing some conference elements with digital elements would not endanger the conference as a whole but open up room for formats that were previously not possible due to the high number of participants. At the same time, virtual participation might also be more accessible for participants and institutions with fewer resources, and be more environmentally sustainable.
- **Short, but intense**: Future BISE conferences could become shorter events in terms of physical presence (a typical BISE conference with all pre- and post-workshops, doctoral consortium etc. used to last about 5 days) because some elements of the conference are now offered online prior to the conference. The focus at the conference venue is on facilitating personal exchanges, networking, and interactions. In this scenario, the basic premise is that typical conference participants in previous years still join in with their physical presence. At the same time, groups that could previously not join (e.g., because of high conference fees or too much travelling effort) may enrich the digital part or the conference experience and discover that the BISE community has a lot to offer.
- **Virtual pure play**: Future BISE conferences may never get back to a format with physical presence because of the substantial CO2 footprint of everyone travelling to a conference venue, high conference fees, modification of reimbursement rules by project sponsors like BMBF, BMWI, or the implications arising from the exclusion of some participants, to name but a few reasons. It is also conceivable that due to new vaccine-resistant mutations of the Covid-19 virus or new pandemics caused by other viruses, bigger events with an international audience are simply not possible for the time being.

Although I have no crystal ball with which to foresee which of these scenarios (or any other scenario) will materialize in the future, I would like to highlight two aspects that – from my point of view – should be considered by future BISE conference organizers:

First, while the (supposed) core value driver of a conference – presenting and discussing research – works very well in a virtual setting, most other aspects mentioned above cannot take place in a virtual setting, or at least may not be replaced with qualitatively comparable digital substitutes. Indeed, many conferences, research seminars, and niche workshops went virtual in the last months. This means that the opportunities to join high quality scientific discourse (typically free of charge) has increased manifold. Consequently, we can expect to see a relative(!) decline in interest in research being presented and discussed at forthcoming BISE conferences. This could be also the trigger to simplify and streamline our comprehensive review process, which I think is possible without the risk of diluting the BISE standard of quality.

Second, both a virtual format and a format with a physical presence each have their own merits, as well as pitfalls. From my experience of the last months, however, hybrid formats invariably fall short of offering not only the combined benefits of both but also of mitigating their respective pitfalls. In my view it would be much preferable to combine these two formats sequentially rather than simultaneously, e.g. by holding a virtual pre-conference, followed by a physical conference, and concluding with an online wrap-up.

Whatever the outcome will be, I very much look forward to the next BISE conference – be it fully virtual, hybrid, or in a purely physical presence.

## Footnotes

1 I use BISE and WI synonymously.

2 www.wi22.eu.
